# Socio-economic determinants of subjective wellbeing toward Sustainable Development Goals: An insight from a developing country

**DOI:** 10.3389/fpsyg.2022.961400

**Published:** 2022-09-14

**Authors:** Anas A. Salameh, Sajid Amin, Muhammad Hassan Danish, Nabila Asghar, Rana Tahir Naveed, Mubbasher Munir

**Affiliations:** ^1^Department of Management Information Systems, College of Business Administration, Prince Sattam Bin Abdulaziz University, Al-Kharj, Saudi Arabia; ^2^Punjab Economic Research Institute, Planning and Development Department, Government of the Punjab, Lahore, Pakistan; ^3^School of Commerce and Accountancy, University of Management and Technology, Lahore, Pakistan; ^4^Division of Management and Administrative Science, Department of Economics, University of Education, Lahore, Pakistan; ^5^Division of Management and Administrative Sciences, University of Education (UE) Business School, University of Education, Lahore, Pakistan; ^6^Department of Economics and Statistics, Dr. Hasan Murad School of Management, University of Management and Technology, Lahore, Pakistan

**Keywords:** income, government effectiveness, social capital, happiness and wellbeing, life satisfaction, life worthwhile, order logit, SDGs

## Abstract

One of the goals of happiness research is to identify the key factors that influence it. Therefore, the present research is designed to examine the determining factors of subjective wellbeing (SWB) in Pakistan. The present research is conducted by collecting the data of 1,566 households in Punjab, Pakistan, using the ordered logit and tobit model. The findings of this research confirm that income, education, government effectiveness, no perceived corruption, and perceived institutional quality improve wellbeing, while lower trust in family and friends, poor health status, living on rent, and dissatisfaction with the services of hospitals lower the level of wellbeing. But individuals with more social ties, who face barriers in health services, live more happily satisfied with their lives. Crime victimization and worrisome terrorism also lower the level of SWB. Findings of research strongly emphasize policymakers and government institutions to improve their quality and take essential measures for improving the governance structure.

## Introduction

Wellbeing is a psychological state of mind that has been studied more over the last 40 years, especially in economics and psychology. This subject got more popular after the measurement of subjective wellbeing (SWB) by Campbell et al. (1976) and Diener (1984). Wellbeing is deliberated as a subjective phenomenon from the point of view that people evaluate themselves by this idea. In a common way, people evaluate the degree of sense of wellness in this aspect. SWB is operationally defined and interpreted as feeling a high level of satisfaction, a higher level of positive affect, and a lower level of negative affect (Deci and Ryan, 2008). One who strongly ratifies these three measures is said to be better in life. Recent past studies have also used the term “happiness” interchangeably for wellbeing. Thus, one’s feeling happier means more wellbeing and vice versa.

The study of SWB has gained wide interest in academia and public policy research worldwide. Developed countries such as Australia, Canada, New Zealand, United Kingdom, and the European regions have started considering maximizing citizens’ “happiness” as their national goal. Even though “happiness studies” has become a popular debate for a long time, they are comparatively new in economics psychology. The advent of serious debates about including happiness in the policy and goals has made the study of potential happiness policies even more vital (Layard, 2006). A vast empirical literature is available on the determinants of happiness and SWB. Now, this matter has attracted the attention of psychologists, economists, and clinical researchers (Helliwell and Putnam, 2004; Kahneman and Krueger, 2006; Deaton, 2008).

Everyone desires to be happy and want to live in a happy and secure community. Happiness is value-seeking as its contagious, spreading like the flu to family, colleagues, friends’ neighbors, and ancestors (Fowler and Christakis, 2008). Individuals benefit from happiness in the form of different varieties, including improved physical, material, and mental health, a longer, retentive, healthier, and improved life, and constructive as well positive interpersonal relations. The objective of international governing organizations’ policies is to maximize the wellbeing of citizens. The Sustainable Development Goals (SDGs) have an international plan aiming to accelerate development until 2030. Improvements in healthcare, education, environmental protection, peace and justice, and institutional excellence are all factors that contribute to SWB (Helliwell and Putnam, 2004; Plagnol, 2011; Ngamaba, 2017; Al Bahar et al., 2021, etc.). According to Dolan et al. (2008), life satisfaction measures are allied with objective factors such as income, education, employment, health status, age, marital status, and other life actions. Quality and equitable education, access to good health services, ownership of financial assets, productive employment, and social protection also promote inclusive development in a country. To achieve these objectives, efforts are being made at national and regional levels to localize and prioritize the SDGs to fulfill SDG strategies and plans as quickly as possible.

Goal 3 of sustainable development is also to promote wellbeing and ensure healthy lives not only among the wealthiest people but also for various strata of society. Goal 4 targets to ensure equitable and quality education. People with middle and high levels of education are more satisfied with their lives than people with low education (Gerdtham and Johannesson, 2001; Shams, 2014; Ngoo et al., 2015). Goal 10 talks about reducing inequalities by enabling and supporting social, governmental, and financial inclusion, increasing pay for the poorest residents, and ensuring social protection and safety rules attain equitable policies. A rise in income level and living standard is also an important indicator of increasing satisfaction among the people of Asia (Dorn et al., 2007; Shams, 2014; Ngoo et al., 2015).

Section “Introduction” of goal 11 aims to ensure gain access to reliable and reasonable accommodation and other essential public services for all. People with home ownership were more satisfied and happier in Great Britain than those living in privately rented houses (Deeming, 2013). Goal 16 of SDGs aims for peace, justice, and effective institutions to minimize conflict and violence, corruption and freedom, and so on. It promotes an inclusive society and institutions to achieve the said targets. To accomplish the SDGs, societies must promote peace and justice. Recent studies also dominate on the effect of governance, violence, institutional quality, and political stability on the level of happiness and life satisfaction (Youssef and Diab, 2021; Ahmadiani et al., 2022).

Considering the significance of human wellbeing, the present research attempts to address the issues and challenges related to the SWB of people in Pakistan. Although studying life satisfaction and happiness has become a popular idea and has gained acute interest among psychologists and economists, there is still a dearth of ideas on this subject. Many studies are available on the study of happiness and life satisfaction which discussed the socio-economic and demographic determinants at the international level (e.g., Clark et al., 2008; Vega, 2016; Ngamaba, 2017; Li and An, 2020; Nizeyumukiza et al., 2020) but very limited context is available on this topic at the national level (Qaisar and Malik, 2015; Jabeen and Khan, 2016; Danish and Khan, 2020, 2021). Thus, the present study is designed to analyze the determinants of SWB in selected districts of Punjab, Pakistan, based on primary data. Factors such as perceived institutional quality, perceived government effectiveness, freedom, social trust, and health services are important to relate to the subjective measures of wellbeing and not discussed earlier at the national level, which will also bridge the gap in this research. Finally, a collective index of measures of SWB will also be a new inclusion in this study. The main aim of this study is to analyze the socio-economics determinants (education, income, living status, debt, employment status, crime, and social capital), demographic determinants (age, gender, marital status, and perceived health), and perceived factors (corruption, institutional quality, government effectiveness, and worrisome on terror) of SWB. The present study will help the policymakers and institutions to work on these factors which improve or lower the level of wellbeing. The rest of the study is designed as follows: Section “Literature review” discusses the previous literature, Section “Data and methodology” is reserved for materials and methods, Section “Results and discussion” highlights the findings of the study, and the study is concluded in Section “Conclusion and policy suggestions.”

### Ranking of happiness in Pakistan

Now we consider life evaluations of respondents in Pakistan, covering the period 2013–2020 (Table 1). These values are taken from the reports of world value surveys.^1^ From 2013 to 2018, Pakistan’s happiness score has risen by 26 points, making it the greatest gainer in the countries of the South Asian Association for Regional Cooperation (SAARC) and internationally one of the top 20 full gainers in 2020 (Helliwell et al., 2020). But it drops down to 4.934 and rank 105 among 149 happiest nations. There might be several reasons for this declining trend. There is a need of the hour to identify those factors that have dropped the happiness level in Pakistan. Therefore, this research aims to identify those factors that increase or decrease the level of SWB. The results of this study would assist policymakers in eliminating or controlling the variables responsible for people’s lower happiness levels.

**TABLE 1:** Ranking of happiness in Pakistan.

Year	Score	Ranking
2013–2015^1^	5.132	92
2015–2017^2^	5.472	75
2016–2018^3^	5.653	67
2017–2019^4^	5.693	66
2018–2020^5^	4.934	105

Source: World Happiness Reports.

^1^Helliwell et al., 2016.

^2^Sachs et al., 2018.

^3^Helliwell et al., 2019.

^4^Helliwell et al., 2020.

^5^Helliwell et al., 2021.

## Literature review

To maximize SWB, it is first necessary to identify the key drivers of wellbeing. Over the last few decades, research into the factors influencing SWB has grown in popularity. Several studies reported that personal characteristics (e.g., age, education, employment status, income, family size, marital status, children, health status, etc.), macroeconomic issues (e.g., inflation, income inequality, unemployment rate, GDP per capita, etc.), and institutional factors (e.g., corruption, government quality, institutional trust, etc.) affect happiness and personal life satisfaction (LS) (e.g., Frey and Stutzer, 2000; Di Tella et al., 2003; Clark et al., 2008; Bjørnskov et al., 2010, etc.).

### Wellbeing and demographics

Demographic determinants of SWB vary according to region, culture, and rank of countries in the previous studies. Age has a U-shaped relationship with happiness and LS in most of the studies (see Frey and Stutzer, 2000; Blanchflower and Oswald, 2004; Helliwell and Wang, 2011; Ngoo et al., 2015), and some studies found the non-linear relationship of age with SWB (Wunder et al., 2013; Proto and Rustichini, 2015). Relationship of family size, number of children, and marital status with SWB have also been considered vastly in previous literature as it affects teenage childbearing, educational attainment, and adult earnings. According to some previous studies, the number of children inversely affect happiness (Di Tella et al., 2003; Alesina et al., 2004; Ferrer-i-Carbonell, 2005; Gwozdz and Sousa-Poza, 2010; Ferreira et al., 2013, etc.), and that married people are happier than single people, who are happier than separated or divorced people (Clark and Oswald, 1994; Peiro, 2006; Dolan et al., 2008).

### Wellbeing with education and wealth

Much evidence is available on the significant and positive impact of income and education on SWB, which is also consistent across countries (e.g., Cuñado and de Gracia, 2012; Pereira and Coelho, 2013, etc.). Similarly, Dorn et al. (2007) also reported the positive impact of income on life happiness in 28 European countries. Alesina et al. (2004) found a positive linear relationship between income and happiness among Americans and European individuals. Results of developing countries are also homogeneous to developed countries. Meyer and Dunga (2014) reported a positive relationship between income and happiness and found that malnourished people are not happy in Africa. Ngoo et al. (2015) used the fifth wave of the Asian Barometer survey and found that the middle-income and high-income groups are happier with their living in comparison to the low-income group, and the results were more robust among the high-income group. Previous studies also suggest that financial wellness is also a major factor in improving wellbeing which is predicted by a high level of income, assets, own house, and low financial stress (Plagnol, 2011), which further increases the level of happiness and LS (Danish and Khan, 2021).

### Wellbeing and social capital

International variations in SWB are based on diverse living circumstances, particularly on the availability of social capital (Helliwell, 2003; Helliwell and Putnam, 2004). According to recent research, social capital is a significant driver of people’s happiness (Chang, 2009; Helliwell and Wang, 2011, etc.). Furthermore, another area of social capital that has gotten much attention is health and wellbeing (Putnam, 2000; Yip et al., 2007; Hoffmann et al., 2019; Danish and Khan, 2020). There is growing evidence of the positive impact of social capital on several aspects of people’s physical and psychological health (e.g., Lomas, 1998; Kawachi et al., 1999; Hawe and Shiell, 2000; Veenstra, 2000; Danish and Khan, 2020). Graham et al. (2011) suggested that having friends improves people’s happiness in Latin America. According to Vega (2016), spending more time with family and friends promotes happiness and improves people’s quality of life (QOL) in Mexico. In another study from Rwanda, Ngamaba (2017) found a positive relationship between trust level and network of friends with happiness. Al Bahar et al. (2021) found a direct association between the relationship with friends and family and happiness among older adults of Abu Dhabi, and Hoogerbrugge and Burger (2018) found a positive relationship between neighborhood social capital and life satisfaction.

### Wellbeing and governance

There are also some variables on the country level that also affect people’s SWB of people like institutional quality, governance, and corruption. Previous literature suggests that political, economic, and judicial institutions are significantly related to the SWB of people at the country level. Trust in police increases nation satisfaction and happiness (Helliwell and Putnam, 2004). A study by Nizeyumukiza et al. (2020) demonstrates the positive and significant relationship between institutional trust and SWB in Indonesia. Youssef and Diab (2021) demonstrate that control of corruption and good governance raises the level of happiness in MENA countries. While on contrary, corruption and poor institutional quality reduce economic opportunity and raise inequality in society which further lead to lower SWB (Rothstein, 2010; Leon et al., 2013). Good governance, in general, decreases inequality and raises happiness (Ott, 2011; Kim and Kim, 2012; Danish and Nawaz, 2022). Crime may also affect the wellbeing of society as a whole and on an individual level. Crime reporting not only affects the victimized individuals but also increases society’s material and immaterial costs.

Determinants of SWB are widely discussed at the international level, but very few studies are available in Pakistan on SWB. For example, Shams (2014) investigated the socio-economic factors of SWB using the data of only 600 rural households from all provinces. Another study by Qaisar and Malik (2015) on public sector workers was conducted to determine their wellness and wellbeing in the context of Islamization. Hasan and Khan (2015) studied happiness as a determinant of the capability of being, functioning, and freedom in life. Jabeen and Khan (2016) empirically analyzed individual happiness in Pakistan by using data from world value surveys, while the effect of income on happiness in Pakistan is found in recent years. Recently, an attempt has been made to analyze the mediating role of health status between social capital and SWB by Danish and Khan (2020), the mediating role of financial satisfaction between income and SWB by Danish and Khan (2021), and the institutional quality and governance by Danish and Nawaz (2022) by using the same data in this research.

The world value survey (WVS) and the GALLUP survey collect data of 1,200 and 1,000 respondents on average from the whole country, which constitutes almost 15 respondents on average from each district, which is also unreliable for policy making at the district level. At the same time, this study will carry out after collecting a sample of more than 1,550 respondents from only four major districts of Punjab. Moreover, few studies use ordinary least square (OLS) regression on an ordinal scale of happiness and life satisfaction. Kim et al. (2015) analyzed the relationship between social capital and life satisfaction in Chinese and Korean elderly immigrants by using OLS, Hoogerbrugge and Burger (2018) also uses OLS while studying the neighborhood-based social capital on life satisfaction in Rotterdam, and Tsuruta et al. (2019) analyzed the relationship between social capital and happiness in Japanese communities and apply OLS with the ordinal outcome of happiness. All these mentioned studies did not apply the appropriate methodology in such kinds of analyses as the appropriate methodology on ordinal outcome dependent variable is order logit or order probit model (Cameron and Trivedi, 2005).

Moreover, no study is found at the national level which analyzes the determinants of SWB, including factors like corruption, governance, institutional quality, social trust, and health services, which play a vital role in improving happiness and life satisfaction among people of any country. In addition, this study also develops an index of three measures of SWB (happiness, LS, and life worthwhile) by using a weighted average method for the index. Thus, the present study fills the gap both at the national and international level in studying SWB, which will be carried through primary data from four major districts of Punjab, Pakistan (Lahore, Faisalabad, Rawalpindi, and Multan).

## Data and methodology

### Data and model

The present study is carried out by collecting the data of 1,566 households’ individuals in four major districts of Punjab, Pakistan (Lahore, Faisalabad, Rawalpindi, and Multan). The data are collected according to the population proportion of each district, randomly considering both rural and urban areas. More than 74% of respondents are men, while the remaining 25–26% are women. Respondents were asked to fill/tell the required information in the questionnaire. More than 1,000 respondents were interviewed from July 2018 to January 2019. While for the remaining 500 respondents, more than 900 questionnaires were distributed through the survey team with a response rate of 60%. The questionnaire was reviewed by three experts in this field, and data were collected after incorporating suggestions of expertise. The questionnaire includes information about household respondents’ personal and demographic characteristics: Economic factors of individuals and households like income, dwelling, debt, and so on, health and perceived qualities and satisfaction with institutions, perceived measures of life-influencing SWB like freedom, crime victim, and safety measures, and some variables of social capital, institutional quality, and perceived government effectiveness.

The following model is finalized to carry out this research.


SWB=i*δ0+δ1i.agei+δ2i.genderi+δ3i.educationi

$$+\delta_{4}\,i.e\,m\,p\,l\,o\,y\,m\,e\,n\,t_{i}+\delta_{5}\,i.M\,S_{i}+\delta_{6}\,i.c\,h\,i\,l\,d\,r\,e\,n_{i}+\delta_{7}\,i.E\,I\,I\,R_{i}$$

$$+\delta_{8}i.residence_{i}+\delta_{9}i.agri._{i}+\delta_{10}i.loan_{i}+\delta_{11}i.HS_{i}$$

$$+\delta_{12}\,i.S\,O\,G\,H_{i}+\delta_{13}\,I.S\,O\,P\,H_{i}+\delta_{14}\,S\,W\,H_{i}+\delta_{15}\,D\,H\,S_{i}+\delta_{16}\,I\,Q\,I_{i}$$

$$+\delta_{17}GEI_{i}+\delta_{18}COR._{i}+\delta_{19}i.Pol._{i}+\delta_{20}i.crime_{i}+\delta_{21}SM_{i}$$

$$+\delta_{22}Freedom_{i}+\delta_{23}WOT_{i}+\delta_{24}Fam.T_{i}+\delta_{25}Nb.T._{i}$$

(1) $$+\delta_{26}FrT._{i}+\delta_{27}PT_{i}+\delta_{28}NOF+\delta_{29}Mem._{i}$$

where, *i* = 1, 2, 3,….., *k* “individuals.”

SWB_i_* is the vector of subjective wellbeing measures [happiness, life satisfaction, life worthwhile, and an index of all three measures subjective wellbeing index (SWBI)]. Variables in Eq. 1 are defined in Table 2 along with sources.

**TABLE 2:** Variables description and sources.

Variable name	Sample items	Scales	References
SWB (EVALUATION MEASURE)	How much satisfy you are with your life as a whole these days?	0–10	Dolan and Metcalfe, 2012; Deeming, 2013
SWB (EXPERIENCE MEASURE)	How much happy you are with your life as a whole these days?	0–10	Dolan and Metcalfe, 2012; Deeming, 2013
SWB (EUDEMONIC MEASURE)	How much you feel worthwhile with your life as a whole these days?	0–10	Dolan and Metcalfe, 2012; Deeming, 2013
AGE	Range	1–5	Di Tella et al., 2003; Meyer and Dunga, 2014, etc.
EMPLOYMENT STATUS	Part-time/full time, etc.	1–5	Ngamaba, 2017
EDUCATION LEVEL	No education to higher education	1–9	Deeming, 2013; Ngamaba, 2017, etc.
MARITAL STATUS	Single, married, etc.	1–4	Gerdtham and Johannesson, 2001; Ngoo et al., 2015
DWELLING	Own or rent	0 or 1	Plagnol, 2011
NO. OF CHILDREN	No child to more than four children	0–4	Di Tella et al., 2003; Shams, 2014, etc.
EQUALIZED INCOME INDEX	15001–30000……>60000	1–5	Di Tella et al., 2003; Helliwell, 2003, etc.
HEALTH STATUS	Very good to very poor	1–5	Helliwell, 2003; Allin and Masseria, 2009; Qaisar and Malik, 2015, etc.
SATISFACTION OF HOSPITAL SERVICES	Competency, treatment, etc.	1–5	WVS
SOCIAL CAPITAL	Trust level and memberships	1–4	Helliwell and Putnam, 2004
CRIME VICTIM	Family and respondent	0 or 1	WVS, Davies and Hinks, 2010
WAR AND TERRORISM	Worriness about terrorism and war against it	1–4	WVS, Clark et al., 2008
POLITICS	Very interested to not at all	1–4	WVS
INSTITUTIONAL QUALITY INDEX	Police, judiciary, and public institutions	1–4	Helliwell and Putnam, 2004
FREEDOM	Not at all to great deal	0–10	WVS
GOVERNMENT EFFECTIVENESS	Very good to very poor	1–5	WVS
PERCEIVED CORRUPTION	Yes or no	1 or 0	WVS
AREA	Rural or urban	0 or 1	Shams, 2014
AGRI LAND	No or Yes	0 or 1	Plagnol, 2011

According to previous research, SWB is defined as “a person’s cognitive judgment of life satisfaction, as well as their feelings and emotions, which economics and psychologists refer to as ‘happiness”’ (Helliwell and Putnam, 2004; Helliwell, 2006; Sarracino, 2013). But there is very little argument on measuring SWB through life worthwhile. Therefore, considering Dolan and Metcalfe (2012) and Deeming (2013), who advocated gaging each of the “Eudemonic” (Worth-While), “Evaluation” (Life Satisfaction), and “Evaluative” (Happiness) measures separately, this study also suggests gaging each of these measures. Following the WVS, respondents are rated on an ordinal scale from 0 (not at all happy/satisfied/worthwhile) to 10 (extremely happy/satisfied/worthwhile) for all aspects of SWB. Moreover, for individual measurement of SWB attributes, a SWBI is also constructed for everyone. Weight for happiness, LS, and worthwhile is assigned to the response of everyone, calculated from the sum values of each measure, and then this weight is multiplied by the actual response of an individual. Finally, all calculated happiness, LS, and worth values are added to construct the SWBI.


(2) $$W=\frac{S\,W\,B}{\sum_{i=1}^{k}S\,W\,B}$$

(3) S=i1n∑i=1kWi*SWBi

(4) SWBI=S+1S+2S3

Finally, the following weights are assigned according to calculations estimated in Eq. 3 to get the SWBI.


(5) S⁢W⁢B⁢I=0.33×Happiness+0.34×L⁢S+0.33×Worthwhile

### Methodology

The objective of this study is to analyze the determinants of SWB. As happiness, life satisfaction, and worthwhile of life have ordinal outcomes (0,1,2,…,10), ordered logistic regression is used to estimate all the models of wellbeing. The order logit model describes an unobserved latent variable (Cameron and Trivedi, 2005). The starting point is an index model with a single latent variable.


(6) $$y_{i}^{*}=\beta \,x_{i}^{\prime }+u_{i}$$

where “*x*” here does not include an intercept, with values of *y*_*i*_ = 0, 1, 2,…,10, being observed as $y_{i}^{*}$ crosses gradually higher thresholds, which are also parameters to be estimated. An ordered logit model arises when *u_i* is logistic (or standard normal) distribution. Moreover, our analyses use the bootstrap standard errors with a maximum of 100 replications. It may be used to get cluster-robust standard errors in cases where clustering causes correlation within a cluster but has no effect on estimator consistency (Cameron and Trivedi, 2005).

The present research has also constructed the SWBI and considered for the analyses to analyze its determinants. SWBI is the discrete variable with limited values (0–10). Therefore, the tobit model is the suitable methodology for the model of SWBI (Cameron and Trivedi, 1986).

## Results and discussion

### Descriptive statistics

Table 3 shows the descriptive statistics for the sample study. The women are 26% of the sample, while the men are 74%. The average age of the respondent is 39 years, with an average education of 12 years and a family size of 6.46. Nearly 56% of sample respondents have an adjusted per-capita family income of less than Rs. 30,000 on average, while just 24% have an average per-capita household income of more than Rs. 45,000. Up to 43.5% of respondents said their health was good, while 5.7 and 1.9% said their health was poor or extremely poor. Only a small percentage of respondents were satisfied with the police, judiciary, and other government institutions. The range for “very high satisfaction” is 6–16%. Only about 16% of respondents believe that governmental institutions are free of corruption. Mean happiness, life satisfaction, and worthwhile were recoded as 6.59, 6.84, and 6.70, respectively, of the sample respondents.

**TABLE 3:** Descriptive analyses.

Variables	Frequency	Proportion (%)
**GENDER (*N* = 1566)**		
Male	1162	74.2
Female	404	25.8
**AGE (*N* = 1566)**		
Up to 25	221	14.1
26–35	527	33.7
36–45	331	21.1
46–55	341	21.8
Above 55	146	9.3
**MARITAL STATUS (*N* = 1566)**		
Single	445	28.4
Married	1070	68.3
Widowed	41	2.6
Others	10	0.6
**EDUCATION (*N* = 1566)**		
No formal education	71	4.5
Primary or below	82	5.2
Secondary	115	7.3
Matric	219	14.0
Intermediate	174	11.1
Bachelor	311	19.9
Masters	403	25.7
Mphil or above	160	10.2
Others	31	2.0
**EMPLOYMENT STATUS (*N* = 1566)**		
Full time	821	52.4
Part time	135	8.6
Self employed	313	20.0
Retired	73	4.7
Unemployed/housewife	224	14.3
**INCOME (*N* = 1566)**		
Up to 15000	334	21.3
15001–30000	540	34.5
30001–45000	302	19.3
45001–60000	138	8.8
>60000	252	16.1
**SELF-REPORTED HEALTH (*N* = 1566)**		
Very good	384	24.5
Good	681	43.5
Fair	382	24.4
Poor	90	5.7
Very poor	29	1.9
**SATISFACTION WITH SERVICES OF GOVERNMENT HOSPITAL (*N* = 1202)**		
Very much	94	7.8
Somewhat	297	24.7
Neutral	328	27.3
Not much	306	25.5
Not at all	177	14.7
**SATISFACTION WITH SERVICES OF PRIVATE HOSPITAL (*N* = 1201)**		
Very much	312	26.0
Somewhat	423	35.2
Neutral	295	24.6
Not much	101	8.4
Not at all	70	5.8
**SATISFY WITH JUDICIARY (*N* = 1566)**		
Not at all	356	22.7
Not much	405	25.9
Somewhat	550	35.1
Very much	255	16.3
**SATISFY WITH POLICE (*N* = 1566)**		
Not at all	662	42.3
Not much	518	33.1
Somewhat	335	21.4
Very much	51	3.3
**SATISFY WITH OTHER PUBLIC INSTITUTIONS (*N* = 1566)**		
Not at all	278	17.8
Not much	511	32.6
Somewhat	671	42.8
Very much	106	6.8
**PERCEIVED CORRUPTION (*N* = 1566)**		
No	1318	84.2
Yes	248	15.8
**MEMBERSHIPS (*N* = 1566)**		
No	712	45.5
Yes	854	54.5
**TRUST ON FAMILY (*N* = 1564)**		
Trust completely	1311	83.8
Trust somewhat	165	10.5
Not very much trust	51	3.3
Not at all trust	37	2.4
**TRUST ON NEIGHBORS (*N* = 1562)**		
Trust completely	389	24.9
Trust somewhat	761	48.7
Not very much trust	318	20.4
Not at all trust	94	6.0
**TRUST ON FRIENDS (*N* = 1563)**		
Trust completely	517	33.1
Trust somewhat	686	43.9
Not very much trust	253	16.2
Not at all trust	107	6.8
**TRUST ON PEOPLE (*N* = 1559)**		
Trust completely	98	6.3
Trust somewhat	241	15.4
Not very much trust	538	34.4
Not at all trust	682	43.6

Source: Author’s Calculations.

Eighty-three percent of respondents completely trust family, while this ratio is only 24 and 33% for neighbors and friends, respectively. The ratio of trust in other people is very low; almost 78% of respondents do not trust people, which shows the lack of confidence in other people and a low level of social capital in Punjab. Only 7.8% of people are satisfied with the services of government hospitals, which indicates the poor performance or management in government hospitals. Therefore, the government must ensure the equity of health services in government hospitals.

**FIGURE 1:**
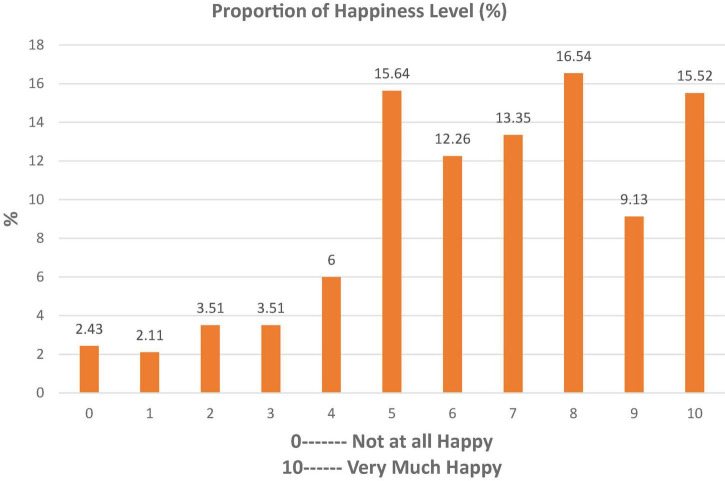
Respondents’ proportion of happiness level.

**FIGURE 2:**
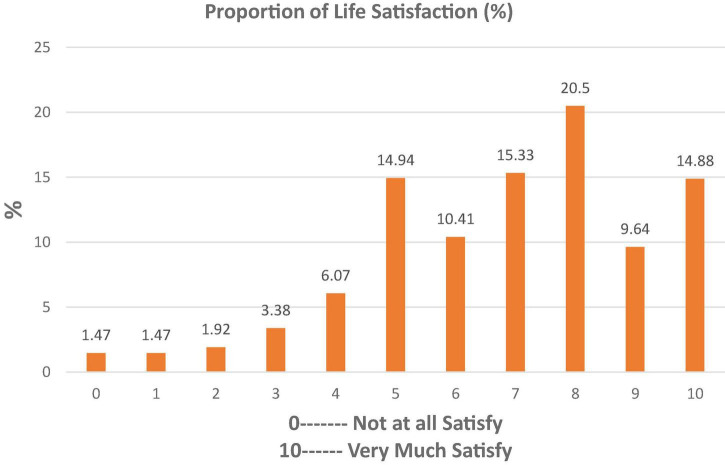
Respondents’ proportion for life satisfaction.

**FIGURE 3:**
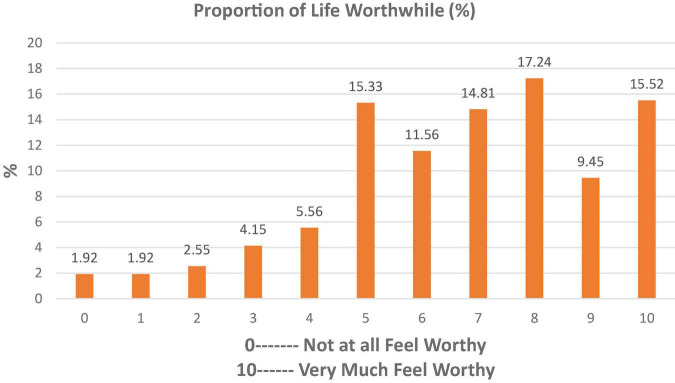
Respondents’ proportion for life worthwhile.

Estimated outcomes of reported SWB among sample respondents (*n* = 1,566) are presented in Figures 1–3. About 8% of sample respondents reported a happiness level of only up to 20% (0–2), 33% reported from 40 to 60% (4–6), and 39% of sample respondents showed a happiness level between 70 and 90% (7–9), while 15.52% of sample respondents were completely satisfied with their lives.

According to Figure 1, most of the respondents were happy with respect to happiness level. Figure 2 shows that the life satisfaction of the respondents was observed high as compared with the average. So, the people of Pakistan are much happier than the average.

Figure 3 highlighted the facts about life worthwhile that show more percentage lie above the average proportion for life worthwhile.

### Regression results

This section presents the results of model 1, which is developed to estimate the determinants of SWB among households in Pakistan. Hierarchical order logit and tobit regressions are applied to analyze the effects of variables on SWB. Column 1 represents the variables used in the regression from Tables 4–8. In contrast, odd ratios for the model of happiness, LS, and worthwhile are reported from columns 2–4, respectively. The last column reports the coefficient values of the tobit model for the determinants of the SWBI. In the first step, only demographic variables are included in the analyses (model Ia). Variables regarding household finances are regressed over SWB in model Ib. Variables regarding the health status of individuals and institutional level variables are added in model Ic. In model Id, variables regarding some personal life experiences and perceptions regarding life are added. Variables of social capital are added in the model Ie. While in the last step, all the variables are added to the model as discussed in Eq. 1 (See Supplementary Appendix).

**TABLE 4:** Demographic determinant of subjective wellbeing method: Ordered logit and tobit [only for subjective wellbeing index (SWBI)].

Variables	Happiness	Life satisfaction	Worth while of life	SWBI
	
	Odd ratios	Odd ratios	Odd ratios	Coeff. values
**GENDER: FEMALE**	1.11	0.95	1.09	0.08
	(0.13)	(0.13)	(0.13)	(0.11)
**AGE**				
26–35	0.62***	0.77*	0.72**	−0.40**
	(0.09)	(0.12)	(0.10)	(0.20)
36–45	0.54***	0.77	0.71	−0.45*
	(0.11)	(0.14)	(0.16)	(0.27)
46–55	0.51***	0.93	0.92	−0.26
	(0.12)	(0.18)	(0.20)	(0.27)
>55	0.45***	0.82	0.79	−0.45
	(0.10)	(0.22)	(0.20)	(0.31)
**EDUCATION**				
Primary or below primary	1.05	1.47	1.20	0.30
	(0.36)	(0.49)	(0.43)	(0.39)
Secondary or below	1.25	2.14**	1.24	0.50
	(0.37)	(0.69)	(0.34)	(0.37)
Matric (10 years)	2.16***	2.29**	2.09***	1.06***
	**(0.59)**	**(0.76)**	**(0.54)**	**(0.29)**
Intermediate (12 years)	3.76***	4.07***	2.64***	1.62***
	(1.05)	(1.36)	(0.73)	(0.33)
Bachelor (14 years)	4.29***	4.05***	2.69***	1.71***
	(1.25)	(1.23)	(0.65)	(0.33)
Masters (16 years)	5.60***	4.54***	3.50***	2.07***
	(1.51)	(1.37)	(0.91)	(0.29)
Mphil or above	6.32***	4.33***	3.67***	2.07***
	(1.87)	(1.39)	(0.97)	(0.34)
Certification/engineer/medical	7.76***	8.15***	7.59***	2.82***
	(3.14)	(3.09)	(3.05)	(0.44)
**EMPLOYMENT**				
Part time employee	0.52***	0.68*	0.73*	−0.64***
	(0.09)	(0.14)	(0.13)	(0.19)
Self-employed	1.59***	1.34**	1.39**	0.50***
	(0.20)	(0.16)	(0.20)	(0.16)
Retired	1.35	1.60	1.01	0.23
	(0.43)	(0.54)	(0.31)	(0.33)
Unemployed	0.97	1.07	0.90	−0.06
	(0.14)	(0.16)	(0.14)	(0.15)
**MARITAL STATUS**				
Married	1.74**	2.05**	1.21	0.72*
	(0.49)	(0.74)	(0.40)	(0.37)
Widowed	1.13	1.27	1.05	0.24
	(0.46)	(0.59)	(0.50)	(0.49)
Divorce	0.72	0.57	0.65	−0.39
	(0.47)	(0.35)	(0.33)	(0.82)
**CHILDREN**				
One child	1.04	1.35	1.13	0.19
	(0.28)	(0.34)	(0.34)	(0.26)
2 children	0.91	1.02	0.69	−0.18
	(0.21)	(0.28)	(0.16)	(0.20)
3 children	0.84	0.91	0.78	−0.24
	(0.20)	(0.22)	(0.23)	(0.24)
4 or more	1.09	0.87	0.77	−0.13
	(0.23)	(0.21)	(0.23)	(0.24)
Constant				4.97***
				(0.51)
Log likelihood	−3336.84	−3265.20	−3348.61	−3286.27
Wald CHI2	338.55	198.68	297.87	302.88
*P* > CHI2	0.0000	0.0000	0.0000	0.0000
Observations	1566	1566	1566	1566

“***,” “**,” and “*” denote the significance level at 1, 5, and 10%, respectively, while SE is given in parenthesis.

**TABLE 5:** Factors of household economy as determinants of subjective wellbeing method: Ordered logit model Ib.

Variables	Happiness	Life satisfaction	Life worthwhile	SWBI
	
	Odd ratios	Odd ratios	Odd ratios	Coeff. values
Residence: rent	0.85	0.76**	0.96	−0.17
	(0.12)	(0.10)	(0.10)	(0.14)
Agri-land: yes	1.29***	1.41***	1.16	0.23**
	(0.12)	(0.16)	(0.12)	(0.10)
Loan: yes	0.80*	0.69**	0.90	−0.24
	(0.09)	(0.11)	(0.13)	(0.16)
**EQUALIZED INCOME INDEX**				
15001–30000	3.19***	2.13***	2.10***	1.17***
	(0.42)	(0.26)	(0.29)	(0.14)
30001–45000	4.90***	2.83***	2.76***	1.63***
	(0.68)	(0.40)	(0.42)	(0.15)
45001–60000	7.90***	4.41***	3.68***	2.14***
	(1.52)	(0.80)	(0.62)	(0.18)
>60000	8.81***	4.65***	3.95***	2.17***
	(1.50)	(0.71)	(0.67)	(0.15)
LOC	0.51***	0.76	0.66**	−0.58**
	(0.10)	(0.15)	(0.11)	(0.26)
Constant				6.10***
				(0.30)
Log likelihood	−3288.76	−3239.00	−3340.52	−3255.60
Wald CHI2	289.52	175.13	158.08	415.61
*P* > CHI2	0.0000	0.0000	0.0000	0.0000
Observations	1566	1566	1566	1566

“***,” “**,” and “*” denote the significance level at 1, 5, and 10%, respectively, while SE is given in parenthesis.

**TABLE 6:** Factors of health and institutions as determinants of subjective wellbeing method: Ordered logit model Ic.

Variables	Happiness	Life satisfaction	Worth-while	SWBI
	
	Odd ratios	Odd ratios	Odd ratios	Coeff.
DHS	0.93	0.94	1.05	0.01
	(0.05)	(0.05)	(0.06)	(0.06)
FDHS	1.36***	1.45***	1.25**	0.31***
	(0.15)	(0.18)	(0.13)	(0.05)
**SOGH**				
Somewhat	0.84	1.08	0.75	−0.15
	(0.22)	(0.23)	(0.23)	(0.24)
Neutral	0.75	1.09	0.82	−0.15
	(0.18)	(0.27)	(0.23)	(0.24)
Not much	0.72	0.89	0.67	−0.30
	(0.18)	(0.21)	(0.21)	(0.25)
Not at all	0.52***	0.86	0.46**	−0.76***
	(0.13)	(0.24)	(0.15)	(0.26)
**SOPH**				
Somewhat	0.81**	0.60***	0.69***	−0.45***
	(0.08)	(0.08)	(0.09)	(0.15)
Neutral	0.61***	0.46***	0.54***	−0.80***
	(0.08)	(0.07)	(0.07)	(0.17)
Not much	0.64**	0.68*	0.82	−0.47**
	(0.12)	(0.14)	(0.17)	(0.23)
Not at all	0.49***	0.41***	0.63**	−0.95***
	(0.13)	(0.09)	(0.14)	(0.27)
SWH	1.11**	1.13***	1.11**	0.15***
	(0.05)	(0.05)	(0.05)	(0.05)
**HEALTH STATUS**				
Good	0.54***	0.47***	0.55***	−0.72***
	(0.08)	(0.06)	(0.08)	(0.13)
Fair	0.27***	0.29***	0.38***	−1.39***
	(0.04)	(0.04)	(0.06)	(0.15)
Poor	0.13***	0.18***	0.23***	−2.15***
	(0.03)	(0.04)	(0.07)	(0.24)
Very poor	0.03***	0.06***	0.13***	−3.49***
	(0.01)	(0.02)	(0.06)	(0.40)
Institutional quality	1.09*	1.09	1.09*	0.13**
	(0.05)	(0.06)	(0.05)	(0.05)
Government effectiveness	1.08	1.07	1.03	0.10*
	(0.05)	(0.05)	(0.05)	(0.05)
Corruption: no	1.48***	1.52***	1.38***	0.48***
	(0.18)	(0.18)	(0.17)	(0.14)
Constant				8.28***
				(0.25)
Log likelihood	−3226.70	−3128.72	−3250.54	−3147.74
Wald CHI2	373.04	526.17	219.93	466.73
*P* > CHI2	0.0000	0.0000	0.0000	0.0000
Observations	1545	1545	1545	1545

“***,” “**,” and “*” denote the significance level at 1, 5, and 10%, respectively, while SEs are given in parenthesis.

**TABLE 7:** Perceived factors of individuals as determinants of subjective wellbeing method: Ordered logit model Id.

Variables	Happiness	Life satisfaction	Worth-while	SWBI
	
	Odd ratios	Odd ratios	Odd ratios	Coeff. values
Political interest somewhat	1.08	1.05	0.93	0.07
	(0.11)	(0.14)	(0.10)	(0.13)
Not much	1.14	0.94	0.76**	0.00
	(0.15)	(0.12)	(0.09)	(0.14)
Not at all	0.66***	0.71*	0.53***	−0.60***
	(0.10)	(0.13)	(0.07)	(0.19)
Crime: yes	1.01	0.75**		
	(0.11)	(0.10)		
Freedom	1.49***	1.61***	1.53***	0.44***
	(0.04)	(0.05)	(0.05)	(0.02)
Security adaptation	0.87***	0.87***	0.86***	−0.20***
	(0.04)	(0.03)	(0.04)	(0.04)
Worrisome on terrorism	0.94*	0.91**	1.03	−0.07**
	(0.04)	(0.04)	(0.04)	(0.04)
Constant				3.86***
				(0.19)
Log likelihood	−3254.60	−3082.15	−3181.99	−3133.95
Wald CHI2	350.63	294.29	295.95	503.77
*P* > CHI2	0.0000	0.0000	0.0000	0.0000
Observations	1566	1566	1566	1566

“***,” “**,” and “*” denote the significance level at 1, 5, and 10%, respectively, while SEs are given in parenthesis.

**TABLE 8:** Factors of social capital determinant of subjective wellbeing method: Ordered logit model Ie.

Variables	Happiness	Life satisfaction	Worth-while	SWBI
	
	Odd ratios	Odd ratios	Odd ratios	Coeff. values
Friends	1.34**	1.43***	1.27*	0.25**
	(0.16)	(0.17)	(0.16)	(0.11)
Memberships: yes	1.67***	1.27***	1.22**	0.48***
	(0.14)	(0.09)	(0.12)	(0.10)
**FAMILY TRUST**				
Somewhat	0.62***	0.77*	0.64***	−0.53***
	(0.10)	(0.11)	(0.09)	(0.18)
Not much	0.41***	0.38***	0.53**	−1.15***
	(0.11)	(0.12)	(0.16)	(0.25)
Not at all	0.34***	0.55	0.50*	−1.14**
	(0.12)	(0.21)	(0.20)	(0.46)
**NEIGHBOR’S TRUST**				
Somewhat	0.98	0.74**	0.82	−0.09
	(0.16)	(0.10)	(0.10)	(0.15)
Not much	1.05	0.84	0.96	0.05
	(0.20)	(0.12)	(0.13)	(0.20)
Not at all	1.33	0.82	0.95	0.07
	(0.31)	(0.20)	(0.26)	(0.27)
**FRIEND’S TRUST**				
Somewhat	0.66***	0.64***	0.64***	−0.55***
	(0.08)	(0.06)	(0.06)	(0.12)
Not much	0.37***	0.38***	0.35***	−1.32***
	(0.06)	(0.07)	(0.06)	(0.20)
Not at all	0.33***	0.35***	0.34***	−1.37***
	(0.08)	(0.12)	(0.11)	(0.28)
**PEOPLE’S TRUST**				
Somewhat	0.94	1.07	0.87	−0.10
	(0.23)	(0.29)	(0.19)	(0.26)
Not much	0.78	1.00	0.90	−0.17
	(0.18)	(0.23)	(0.23)	(0.24)
Not at all	0.79	0.99	0.99	−0.15
	(0.17)	(0.27)	(0.24)	(0.25)
Constant				7.29*** (0.24)
Log likelihood	−3269.20	−3171.76	−3259.87	−3199.62
Wald CHI2	170.39	170.00	205.11	180.93
*P* > CHI2	0.0000	0.0000	0.0000	0.0000
Observations	1537	1537	1537	1537

“***,” “**,” and “*” denote the significance level at 1, 5, and 10%, respectively, while SEs are given in parenthesis.

Results of demographic and personal characteristics are presented in Table 4, where age, gender, education, employment, marital status, and number of children are regressed over SWB. Contrary to the literature, age is negatively and significantly related to the happiness level. Older people above the age of 55 years reported the least level of happiness, while younger age people reported a high level of happiness. The odds of being happy at the upper age group level are less by 0.55 (OR = 0.45; *p* < 0.01) than the age group of people up to 25 years and by 0.17 than the age group of 26–35 years. Most of the aged people perceived poor health status which might be the reason for a low level of happiness. While, no significant relationship between age with LS and worthwhile and inclusive with overall wellbeing, as only the middle age group is significantly related to SWBI but not robust. Campbell et al. (1976) also predicted that demographic factors (age, gender, education, and marital status) only account for a 20% variation in SWB. The level of education is also significantly related to all measures of SWB. Individuals with higher education levels are happier, satisfied, and feel more worthfull about their lives compared to a very low level. There is no difference in the SWBI of university graduates as masters and above-level educated people have the same level of SWBI (β = 2.07; *p* < 0.01). Individuals holding certificates of accountancy or degrees in medical or engineering reported the highest level of SWB, and their happiness increased by odds of 1.16 (OR = 7.76; *p* < 0.01) to those holding other master-level degrees. Education improves the quality of thinking thus individual’s probability to get a job also increases which further raises the level of income which is directly proportional to happiness and LS (Ngoo et al., 2015).

Similarly, LS and worthwhileness of these people are higher by odds of 3.61 and 4.09 than the reference group (16-year education). Previous studies also find that higher education robustly affects the level of happiness and overall life satisfaction (Frey and Stutzer, 2000; Alesina et al., 2004; Jongbloed, 2018), while primary and secondary education have the least effect on SWB or sometimes have no effect (Davies and Hinks, 2010; Jongbloed, 2018; Nizeyumukiza et al., 2020). In Pakistan, it might be reasoning that highly educated people tend to get more employment or start their businesses to achieve a minimum standard of life to remain happy. Employment status also affects the SWB of people. Results of the present study also indicate that self-employed people are happier by odds of 0.59 than full-time employees, which are happier by odds of 0.48 than part-time employees. Overall, the wellbeing of self-employed people is high. At the same time, other groups (retired, unemployed/housewives) are not significantly related to SWB. The results of this study are also in line with previous studies and our hypothesis that self-employed and full-time employed people are happier with their lives (Headey and Wooden, 2004; Davies and Hinks, 2010), while unemployment negatively affects the happiness of people (Wang and Yang, 2016). Age, marital status, and the number of children are also statistically unrelated to SWB. However, up to three children, SWB decreases and then again increases. While married people are happier and more satisfied with their lives compared to single people, their overall wellbeing is also significantly higher than single. Marriage is generally seen as a strong predictor of wellbeing since it provides emotional, social, and economic support among married couples, hence increasing their happiness (Ndayambaje et al., 2020).

Moreover, women only show less satisfaction with their lives but are happier than men. However, this relationship is not significant at any level. Previous studies also show heterogeneous results on age, sex, and marital status. Few studies show the negative effect of age on happiness (Gwozdz and Sousa-Poza, 2010; Nikolaev and Rusakov, 2016). One of the major reasons for declining happiness levels with age is that health at an older age also tends to be poor in Pakistan, which causes stress and affects mental health.

Table 5 shows that holding agricultural land and a higher income level have a positive and significant impact on SWB. The happiness of the upper-income group (>60,000) is higher by 8.81 odds than the lowest income group (<15,000: reference category), and it is higher by 0.90 odds than the fourth quantile group (45,001–60,000). Similarly, LS of the upper-income group is higher by odds of 2.95, and worthwhile is higher by odds of 2.17 than the reference category (<15,000). Happiness and life satisfaction of agricultural landholders are also on the higher side by odds of 1.29 (OR = 1.29; *p* < 0.01) and 1.41 (OR = 1.41; *p* < 0.05), respectively. The results of this study are also consistent with hypotheses and many previous studies that find a positive impact of income and household assets on SWB (Alesina et al., 2004; Graham, 2011; Brown and Gray, 2016; Knoll and Pitlik, 2016; Krulichová, 2018, etc.). Improving the level of income eases the debt constraints and raises the consumption level by individuals or households which further raises the level of satisfaction and thus increases happiness (Pereira and Coelho, 2013).

On the other side, individuals with an outstanding loan to repay are less happy and satisfied with their lives by odds of 0.20 and 0.31, respectively, than those with no outstanding loan. Moreover, for borrowers who do not feel comfortable or are under financial stress, their SWB level further decreases. Previous studies also show that financial stress causes mental illness and lowers satisfaction with life and happiness (Brown and Gray, 2016; Aripin and Puteh, 2017, etc.). Overall, the SWBI of such people also decreased by 0.58 than the predicted values of SWBI (*p* < 0.05). Finally, people who live in rented houses have lower SWB than those who own their houses, but this relationship is significant only with life satisfaction (OR = 0.76; *p* < 0.05).

Health status, as expected, produces the same results as our hypothesis predicts. Poor health status is negatively related to the SWB (Table 6). The estimated SWBI of individuals with the worst level of self-reported health is at a minimum level (β = −3.42; *p* < 0.01). The reported happiness of individuals with poor health status is estimated to decrease by odds of 0.41 to those who reported good HS (OR = 0.13, *P* < 0.01). The level of LS and worthwhile is also badly affected by individuals’ poor SRH, and this relationship is significant at a 1% level. Poor health status is the major cause of low happiness, and life satisfaction as a person with poor health cannot enjoy the green of life. Moreover, individual with poor health restrict themselves socially which create stress and cause a lowering of level of happiness. The results of our study are also in line with hypotheses and previous studies in which good health is positively related to SWB while poor health is negatively related to SWB (Kahneman and Deaton, 2010; Garrett and James, 2013; Knoll and Pitlik, 2016). Hospitals’ satisfaction and services of hospitals also account for the wellbeing of people who avail or availed of the services when needed. A higher level of satisfaction with the services of hospitals (mostly visited by households) increases the wellbeing of individuals and vice versa. Similarly, people who were not satisfied overall with the situations of government or private hospitals reported a low level of happiness, LS, and worthwhile of life as well.

Moreover, respondents also show a low level of wellbeing if they face difficulties approaching hospitals, but this relationship is not statistically significant with the wellbeing (*p* > 0.1). However, interaction with several friends of barriers to the hospital improves the level of wellbeing, and relationship also becomes significant. In our society, people with strong social capital can easily approach doctors’ services with less cost minimizing the barriers to approaching health services, hence more wellness among people.

The institutional quality index (IQI) is also positively related to the SWB of people. Respondents who reported more satisfaction with police, court, and public institutions are happier and satisfied with their lives. SWBI is positively related to greater satisfaction with IQI (β = 0.41; *p* < 0.01). Similarly, the effective government index is also regressed over happiness and LS, which is also positively related to both factors of SWB, but a significant relationship is only found with LS. A one standard deviation increase in the satisfaction with government quality also increases the level of LS by odds of 1.10 (*p* < 0.05). Individuals who perceived better quality of institutions and government efficacy perceived more trust in such institutions and believe more support from institutions which raises the level of happiness and life worthwhile (Danish and Nawaz, 2022). Many empirical studies have found a strong positive relationship between trust in institutions and happiness. Dolan et al. (2008) suggested an important link between trust of association and wellbeing by conducting a literature review on SWB. Evidence from underdeveloped nations also suggests a positive correlation between institutional trust and SWB (Nizeyumukiza et al., 2020). Finally, respondents who perceived that the government was not involved in the corruption reported more levels of happiness as compared to those who perceived negatively about government corruption (OR = 1.48, *p* < 0.01). Tay et al. (2014) used data from a Gallup World Poll survey of 150 nations to find that perceived corruption lowers national GDP, lowering LS and pleasant feelings. Controlling corruption, on the other hand, raises national happiness (Li and An, 2020). Corruption has become a major problem that has affected the institutional trust in Pakistan as it also lowers the tax collection to help the poor and maintain the public health system (Habibov et al., 2019) which may reduce the level of SWB. While on the contrary, intuitional trust increases the willingness to pay more taxes by society, which not only increases the wealth of the nation but also increases government spending on the public which increases the level of happiness in the long term (Habibov et al., 2018).

Some variables other than the socio-economic factors may affect the psychological and mental state of happiness and life satisfaction. Some of these factors are also regressed over SWB, and the results are presented in Table 7. Results of the study depict that freedom is one variable that strongly affects an individual’s wellbeing level. More freedom of life choices led to increased happiness by odds of 1.49, LS by odds of 1.61, worthwhile by odds of 1.53, and all are significant at a 1% level (*p* < 0.01). In the past 2 years, crime victimization, either by respondent individual or family, also badly affect the life satisfaction of individuals. Individuals who also adopt some security measures in life, they always psychologically need to prevent risks. Hence, their wellbeing is also negatively affected as reported in our results (OR = 0.87; *p* < 0.01). Over the last decade, terrorism, civil war, and war against terrorism involving the country have also affected the state of people. People are mentally stressed with such situations in the country. Results of our study also confirm the hypothesis that for people who take the stress and worry about such situations in the country, their happiness and LS also decrease by odds of 0.06 and 0.09, respectively. Previous studies also find that self-victimization of crime or family significantly affects the level of happiness (Graham, 2011; Staubli et al., 2014; Krulichová, 2018, etc.).

Table 8 presents the results of social capital as a determinant of SWB. The number of friends, memberships with associations or alike, and strong trust in family and friends are all positively and significantly related to factors of SWB. A one standard deviation increase in the number of friends also increases the happiness level by odds of 1.34, LS by odds of 1.43, and worthwhile by odds of 1.27. It also increases the predicted probability of SWBI by 25%. Similarly, respondents registered with some voluntary organizations or associations more socially interact with other people and feel happier and satisfied with their lives. This relationship is also significant at a 1% level with all measures of SWB.

As discussed in the literature, trust is also an important determinant of SWB. People who trust their family, neighbors, and friends get the reward of trust in return, which connects them more strongly within and outside the groups. Such social engagement brings more happiness and, hence, more satisfaction in their lives. Moreover, the worth of such people also increases when they socially interact with others with the reciprocity of trust. Results of our study also depict that a decrease in the level of trust also decreases the level of wellbeing, but this relationship is significant only with the trust level of family and friends. There is growing evidence that social capital favors people’s physical and psychological health (Lomas, 1998; Kawachi et al., 1999; Veenstra, 2000; Ateca-Amestoy et al., 2014; Tsuruta et al., 2019; Yeo and Lee, 2019, etc.).

## Conclusion and policy suggestions

Societal wellbeing to reach height is becoming a big idea. It has now become the desired campaign, particularly in middle-income countries like Pakistan, to improve the wellbeing of its people. This study has added to a journey of public policy by understanding and developing the framework for SWB after collecting the data from 1,566 households in Punjab, Pakistan. The present research uses three dimensions of SWB (happiness, LS, and Life worthwhile) in addition to an index of all three indicators. The main objective of its study is to explore the factors which increase or lower the level of SWB. The present study uses mix methodology, including the order logit for all three measures of SWB and the tobit model for SWBI. Results of the study show the importance of social capital for improving wellbeing. An increase in social capital may strengthen social support, consequently improving an individual’s wellbeing.

Moreover, income is also the major determinant of SWB. Education, employment status, freedom of choice, satisfaction with the services of hospitals, good governance, and institutional trust are all variables that positively affect happiness and LS. While crime victims, security adaptation measures, poor health, and perceived corruption adversely affect the SWB of people in Pakistan. After the analyses of our model, we believe that the higher the income level, trust in institutions, and improved quality of hospitals and government institutions, the higher will be the SWB. The study found that people’s age is negatively related to SWB in contrast to previous studies due to the poor health status among old individuals. Moreover, individuals who do not own their houses and have outstanding debt show a lower level of wellbeing. Finally, this study demonstrates that married people are more happy than single and divorced and women are happier than men.

Based on the findings, the present study emphasizes improving the level of education and living standard of people and improving the governance and institutions of the country. Although we cannot control these variables, this study can assist the policymakers and institutions to work on those areas and factors which can improve people’s overall wellbeing and psychological health. Policies regarding health for future generations at old age need to be re-emphasized. Such policies should be developed, which would not only improve the stability of good health in old age level but also increase life expectancy. In this stance, old age people who are not getting benefits from the current health schemes should also be included in the road map of health facilities. Moreover, the government can introduce the economic health insurance scheme all over the country developed on the model of Western and European countries.

Presents study also highlights the role of government which is critical for enhancing wellbeing and QOL because it has tools such as organization, money, and public policies that can impact the wellbeing of people. Furthermore, political institutions play a vital role in broadening the distribution of economic and political power, allowing its citizens to open market access and facilitate education and investment opportunities leading to more wellness in society. Furthermore, the study advises policymakers and institutions to cut interest rates to boost investment among lower- and middle-income groups, which may increase their assets and SWB. There should be no barriers and delays in obtaining the health services lacking the social capital. This study also suggests that the government develop housing projects for rented people and provide affordable housing on easy installments equivalent to their rent and register on their names when the rental amount becomes equal to housing value.

The present research is meaningful for public policy and to reshape the government’s plan for people’s wellbeing. This research is limited to four districts of Pakistan due to time and cost constraints but based on valid data from respondents from the population. In the future, this research can be expanded all over the country to provide a broader perspective about SWB with the inclusion of other variables like race, ethnicity, environmental factors, and so on.

## Data availability statement

The original contributions presented in this study are included in the article/supplementary material, further inquiries can be directed to the corresponding author.

## Author contributions

SA and MD conceived the idea of the study. NA, MM, and RN worked on the research methodology and helped in drafting the manuscript. MM worked on the results and analysis and interpretation of model results. NA supervised the project and intensively edited the language of the manuscript. AS and RN approved and read the final manuscript and participated in the critical appraisal of the manuscript. All authors contributed to the article and approved the submitted version.

## Footnotes

1 Values are estimated on 3 years average, and rankings in 2016, 2018, and 2019 are extracted from the reports of perspective years.
