# Supplementary material for: Socio-economic determinants of subjective wellbeing toward Sustainable Development Goals: An insight from a developing country
Source: Front Psychol. 2022 Sep 14;13:961400. doi: 10.3389/fpsyg.2022.961400 (PMC9515650; doi:10.3389/fpsyg.2022.961400)
Supplement: Supplementary file 1 [file Data_Sheet_1.docx]

**APPENDIX (Supplementary file)**

**Determinant of Subjective Well-Being (All Variables Model)**

**Method: Ordered Logit and Tobit for SWBI**

| **Variables** | **Happiness** | **Life Satisfaction** | **Worth- While of Life** | **SWBI** |
| --- | --- | --- | --- | --- |
|  | **Odd Ratios** | **Odd Ratios** | **Odd Ratios** | **Coeff. Values** |
| Gender: Female | 1.23* | 1.07 | 1.18 | 0.20* |
|  | (0.15) | (0.15) | (0.14) | (0.11) |
| **Age** | | | | |
| 26-35 | 0.72** | 1.04 | 0.96 | -0.06 |
|  | (0.12) | (0.19) | (0.18) | (0.15) |
| 36-45 | 0.56*** | 0.90 | 0.81 | -0.19 |
|  | (0.12) | (0.22) | (0.19) | (0.19) |
| 46-55 | 0.58** | 1.24 | 1.16 | 0.00 |
|  | (0.15) | (0.25) | (0.28) | (0.20) |
| >55 | 0.40*** | 0.87 | 0.85 | -0.28 |
|  | (0.11) | (0.26) | (0.23) | (0.24) |
| **Education** | | | | |
| primary or below primary | 0.90 | 1.59* | 1.08 | 0.10 |
|  | (0.32) | (0.41) | (0.43) | (0.27) |
| Secondary or below | 0.78 | 1.51 | 0.73 | -0.07 |
|  | (0.23) | (0.54) | (0.24) | (0.25) |
| Matric  (10 Years) | 1.32 | 1.63** | 1.21 | 0.29 |
|  | (0.38) | (0.40) | (0.39) | (0.24) |
| Intermediate  (12 Years) | 1.92** | 2.49*** | 1.26 | 0.65*** |
|  | (0.56) | (0.65) | (0.38) | (0.25) |
| Bachelor  (14 Years) | 1.72* | 2.18*** | 1.12 | 0.50** |
|  | (0.49) | (0.59) | (0.35) | (0.24) |
| Masters  (16 Years) | 1.87** | 2.07** | 1.47 | 0.66*** |
|  | (0.57) | (0.60) | (0.47) | (0.24) |
| MPhil or Above | 2.08** | 2.14*** | 1.59 | 0.71*** |
|  | (0.72) | (0.63) | (0.57) | (0.27) |
| Certification / Engineer/ Medical | 2.90** | 4.54*** | 3.46*** | 1.49*** |
|  | (1.20) | (1.71) | (1.53) | (0.37) |
| **Employment** | | | | |
| Part Time Employee | 0.52*** | 0.70* | 0.73 | -0.55*** |
|  | (0.10) | (0.14) | (0.17) | (0.16) |
| Self-Employed | 1.11 | 0.98 | 1.06 | 0.06 |
|  | (0.16) | (0.14) | (0.16) | (0.12) |
| Retired | 0.98 | 1.10 | 0.71 | -0.15 |
|  | (0.31) | (0.35) | (0.21) | (0.23) |
| Unemployed | 1.18 | 1.34 | 1.18 | 0.18 |
|  | (0.21) | (0.26) | (0.22) | (0.14) |
| **Marital Status** | | | | |
| Married | 2.21*** | 2.45* | 1.17 | 0.79** |
|  | (0.67) | (1.18) | (0.33) | (0.33) |
| Widowed | 1.38 | 1.33 | 0.90 | 0.36 |
|  | (0.70) | (0.90) | (0.43) | (0.41) |
| Divorce | 1.25 | 0.60 | 0.91 | -0.11 |
|  | (0.87) | (0.43) | (0.51) | (0.60) |
| Residence: Own | 1.03 | 1.00 | 1.20 | 0.09 |
|  | (0.14) | (0.12) | (0.17) | (0.12) |
| Agri-land: Yes | 1.20 | 1.25** | 1.13 | 0.15 |
|  | (0.14) | (0.14) | (0.14) | (0.10) |
| Loan: Yes | 0.67*** | 0.72*** | 0.87 | -0.26** |
|  | (0.09) | (0.08) | (0.10) | (0.11) |
| **Children** | | | | |
| One child | 0.80 | 1.05 | 0.94 | -0.04 |
|  | (0.24) | (0.30) | (0.28) | (0.24) |
| 2 children | 0.95 | 1.12 | 0.72 | -0.07 |
|  | (0.27) | (0.28) | (0.17) | (0.24) |
| 3 children | 0.93 | 1.06 | 0.91 | -0.02 |
|  | (0.25) | (0.28) | (0.22) | (0.23) |
| 4 or more Children | 1.21 | 0.94 | 0.83 | 0.01 |
|  | (0.32) | (0.26) | (0.20) | (0.23) |
| **Income** | | | | |
| 15001-30000 | 2.30*** | 1.60*** | 1.56*** | 0.35 |
|  | (0.40) | (0.29) | (0.25) | (0.35) |
| 30001-45000 | 2.92*** | 1.79*** | 1.72*** | 0.60*** |
|  | (0.52) | (0.36) | (0.30) | (0.12) |
| 45001-60000 | 3.98*** | 2.28*** | 1.92*** | 0.78*** |
|  | (0.91) | (0.51) | (0.48) | (0.15) |
| >60000 | 4.76*** | 2.27*** | 1.91*** | 1.04*** |
|  | (1.01) | (0.54) | (0.43) | (0.18) |
| DHS | 0.97 | 1.02 | 1.19*** | 0.06 |
|  | (0.08) | (0.10) | (0.07) | (0.06) |
| FDHS | 0.97 | 0.96 | 0.79 | -0.09 |
|  | (0.17) | (0.24) | (0.12) | (0.14) |
| **SOGH** | | | | |
| Somewhat | 0.95 | 1.27 | 0.89 | 0.02 |
|  | (0.26) | (0.27) | (0.21) | (0.20) |
| Neutral | 0.71 | 1.12 | 0.89 | -0.13 |
|  | (0.20) | (0.23) | (0.21) | (0.20) |
| Not Much | 0.68 | 0.89 | 0.66 | -0.30 |
|  | (0.19) | (0.18) | (0.17) | (0.20) |
| Not at all | 0.71 | 1.22 | 0.64 | -0.33 |
|  | (0.24) | (0.31) | (0.20) | (0.22) |
| **SOPH** | | | | |
| Somewhat | 0.93 | 0.68*** | 0.76 | -0.25** |
|  | (0.15) | (0.10) | (0.13) | (0.13) |
| Neutral | 0.71** | 0.52*** | 0.60** | -0.51*** |
|  | (0.12) | (0.09) | (0.13) | (0.14) |
| Not Much | 0.81 | 0.81 | 0.93 | -0.19 |
|  | (0.21) | (0.19) | (0.22) | (0.19) |
| Not at all | 0.81 | 0.55* | 0.76 | -0.43* |
|  | (0.22) | (0.19) | (0.24) | (0.22) |
| SWH | 1.09* | 1.09 | 1.05 | 0.10** |
|  | (0.06) | (0.05) | (0.05) | (0.04) |
| **Health Status** | | | | |
| Good | 0.55*** | 0.49*** | 0.57*** | -0.63*** |
|  | (0.09) | (0.07) | (0.08) | (0.11) |
| Fair | 0.31*** | 0.39*** | 0.48*** | -0.99*** |
|  | (0.06) | (0.06) | (0.09) | (0.13) |
| Poor | 0.23*** | 0.28*** | 0.38*** | -1.37*** |
|  | (0.07) | -0.07 | (0.11) | (0.20) |
| Very Poor | 0.11*** | 0.17*** | 0.35** | -1.99*** |
|  | (0.06) | (0.12) | (0.16) | (0.34) |
| Number of Friends | 1.08 | 1.13 | 1.24 | 0.13 |
|  | (0.16) | (0.22) | (0.18) | (0.13) |
| Memberships: Yes | 1.17* | 1.00 | 0.91 | 0.05 |
|  | (0.10) | (0.12) | (0.09) | (0.09) |
| **Family Trust** | | | | |
| Somewhat | 0.67** | 0.88 | 0.76 | -0.33** |
|  | (0.11) | (0.15) | (0.13) | (0.14) |
| Not Much | 0.51* | 0.52* | 0.65 | -0.64*** |
|  | (0.19) | (0.18) | (0.22) | (0.24) |
| Not at all | 0.33** | 0.53* | 0.69 | -0.87*** |
|  | (0.14) | (0.18) | (0.30) | (0.29) |
| **Neighbour’s Trust** | | | | |
| Somewhat | 1.11 | 0.80 | 0.83 | -0.09 |
|  | (0.14) | (0.12) | (0.12) | (0.11) |
| Not Much | 1.10 | 0.89 | 0.98 | -0.02 |
|  | (0.17) | (0.15) | (0.19) | (0.14) |
| Not at all | 1.27 | 0.89 | 0.80 | -0.06 |
|  | (0.34) | (0.25) | (0.19) | (0.21) |
| **Friend’s Trust** | | | | |
| Somewhat | 0.79** | 0.77* | 0.77** | -0.27** |
|  | (0.09) | (0.10) | (0.10) | (0.10) |
| Not Much | 0.53*** | 0.53*** | 0.50*** | -0.77*** |
|  | (0.09) | (0.10) | (0.08) | (0.14) |
| Not at all | 0.52*** | 0.57* | 0.53*** | -0.70*** |
|  | (0.12) | (0.17) | (0.11) | (0.20) |
| **People’s Trust** | | | | |
| Somewhat | 0.84 | 1.09 | 0.87 | -0.15 |
|  | (0.23) | (0.34) | (0.25) | (0.20) |
| Not Much | 0.78 | 1.14 | 1.05 | -0.10 |
|  | (0.19) | (0.28) | (0.27) | (0.19) |
| Not at all | 0.78 | 1.08 | 1.18 | -0.07 |
|  | (0.19) | (0.26) | (0.31) | (0.19) |
| Institutional Quality | 1.04 | 1.02 | 1.03 | 0.06 |
|  | (0.06) | (0.06) | (0.04) | (0.04) |
| Government Effectiveness | 1.05 | 1.10* | 1.04 | 0.05 |
|  | (0.05) | (0.06) | (0.05) | (0.04) |
| Corruption: No | 1.46*** | 1.36** | 1.31** | 0.35*** |
|  | (0.18) | (0.19) | (0.16) | (0.12) |
| **Political Interest** | | | | |
| Somewhat | 1.14 | 1.05 | 0.92 | 0.06 |
|  | (0.17) | (0.15) | (0.11) | (0.12) |
| Not Much | 1.23 | 0.99 | 0.81 | -0.01 |
|  | (0.20) | (0.15) | (0.11) | (0.13) |
| Not at all | 1.08 | 0.90 | 0.62*** | -0.19 |
|  | (0.18) | (0.13) | (0.09) | (0.13) |
| Crime Victim: Yes | 1.00 | 0.80 | - | - |
|  | (0.16) | (0.15) | - | - |
| Freedom | 1.35*** | 1.49*** | 1.45*** | 0.34*** |
|  | (0.04) | (0.05) | (0.04) | (0.02) |
| Security Adaptation | 0.94 | 0.92 | 0.91** | -0.10** |
|  | (0.04) | (0.05) | (0.04) | (0.04) |
| Worrisome about Terrorism | 0.92 | 0.91** | 1.01 | -0.09** |
|  | (0.05) | (0.04) | (0.04) | (0.04) |
| Constant | - | - | - | 4.27*** |
|  | - | - | - | (0.53) |
| Log Likelihood | -2941.36 | -2854.74 | -3000.48 | -2809.75 |
| Wald Chi2 | 1768.72 | 3372.08 | 1440.03 | 999.67 |
| P>Chi2 | 0.0000 | 0.0000 | 0.0000 | 0.0000 |
| Observations | 1537 | 1537 | 1537 | 1537 |

“***”, “**” and “*” denotes the significance level at 1%, 5% and 10% respectively, while Bootstrap Standard Errors are shown in parenthesis.
